# Effects of Adding Four Sessions of Ultrasound-Guided Percutaneous Electrical Nerve Stimulation to an Exercise Program in Patients with Shoulder Pain: A Randomized Controlled Trial

**DOI:** 10.3390/jcm13113171

**Published:** 2024-05-28

**Authors:** Claudia Valenzuela-Rios, José L. Arias-Buría, Jorge Rodríguez-Jiménez, María Palacios-Ceña, César Fernández-de-las-Peñas

**Affiliations:** 1 Escuela Internacional de Doctorado, Universidad Rey Juan Carlos, 28933 Móstoles, Madrid, Spain; cvalenzuelarios@gmail.com; 2 Department of Physical Therapy, Occupational Therapy, Physical Medicine and Rehabilitation, Universidad Rey Juan Carlos (URJC), 28922 Alcorcón, Madrid, Spain; joseluis.arias@urjc.es (J.L.A.-B.); jorge.rodriguez@urjc.es (J.R.-J.); maria.palacios@urjc.es (M.P.-C.)

**Keywords:** shoulder pain, suprascapular nerve, axillar nerve, exercise

## Abstract

**Objective:** Percutaneous electrical nerve stimulation (PENS) appears to be effective for the treatment of musculoskeletal pain. The aim of this trial was to investigate the effects on disability and pain, as well as on the psychological aspects of adding PENS into an exercise program in patients with subacromial pain syndrome. **Methods:** A randomized, parallel-group clinical trial was conducted. Sixty patients with subacromial pain were allocated into exercise alone (n = 20), exercise plus PENS (n = 20), or exercise plus placebo PENS (n = 20) groups. Patients in all groups performed an exercise program twice daily for 3 weeks. Patients allocated to the PENS group also received four sessions of ultrasound-guided PENS targeting the axillar and suprascapular nerves. Patients allocated to the exercise plus placebo PENS received a sham PENS application. The primary outcome was related disability (Disabilities of the Arm, Shoulder, and Hand, DASH). Secondary outcomes included mean pain, anxiety levels, depressive symptoms, and sleep quality. They were assessed at baseline, one week after, and one and three months after. An analysis was performed using intention-to-treat with mixed-models ANCOVAs. **Results**: The results revealed no between-group differences for most outcomes (related disability: F = 0.292, *p* = 0.748, n^2^_p_ = 0.011; anxiety: F = 0.780, *p* = 0.463, n^2^_p_ = 0.027; depressive symptoms: F = 0.559, *p* = 0.575, n^2^_p_ = 0.02; or sleep quality: F = 0.294, *p* = 0.747, n^2^_p_ = 0.01); both groups experienced similar changes throughout the course of this study. Patients receiving exercise plus PENS exhibited greater improvement in shoulder pain at one month than those in the exercise (Δ −1.2, 95%CI −2.3 to −0.1) or the placebo (Δ −1.3, 95%CI −2.5 to −0.1) groups. **Conclusions:** The inclusion of four sessions of ultrasound-guided PENS targeting the axillar and suprascapular nerves into an exercise program did not result in better outcomes in our sample of patients with subacromial pain syndrome at one and three months after treatment.

## 1. Introduction

Shoulder pain represents a significant health problem presenting with a prevalence across different countries of 15% and an incidence from 7.7 to 62 per 1000 persons per year in the general population [1]. The prevalence of shoulder pain is even higher in workers over the age of 50 [2]. The societal burden of shoulder pain is substantial; in Sweden, the annual costs per patient in primary health care was estimated to be EUR 4139 [3], whereas in Denmark, the total annual costs were estimated to be USD 1.21 billion [4].

It seems that the effects of surgical procedures on pain and function is similar when compared with conservative interventions [5]; therefore, conservative treatment is usually the first therapeutic option chosen by most chronic shoulder pain patients. However, no consensus exists on the most appropriate treatment strategy for shoulder pain since evidence supports the application of different interventions such as exercise [6], electrotherapy [7], dry needling [8], and manual therapy [9] for managing this condition, but with heterogeneous levels of evidence. An umbrella review of systematic reviews found strong recommendations for exercise as the first-line treatment and moderate evidence of no effects for electromedical interventions in patients with shoulder pain [10]. This review also found strong recommendations for integrating manual therapy as an additional intervention to exercise [10].

Interestingly, most treatment strategies investigated for shoulder pain focus on soft tissues (muscle, tendon), and although it indirectly affects the peripheral nerve system, direct stimulation of nerve tissues is lacking. Thus, interventions directly targeting nerve tissues have been proposed as potential novel approaches for managing chronic pain [11]. Percutaneous electrical nerve stimulation (PENS) consists of applying an electrical current throughout filiform solid needles targeting a specific tissue (e.g., muscle, ligament, nerve). The term PENS includes several therapeutic approaches. For instance, electrical dry needling or electroacupuncture could be considered PENS interventions since both apply an electrical current through a filiform solid needle; however, the clinical reasoning and technical performance of both approaches are different. Electrical dry needling is clinically based on pain neuroscience (e.g., Western medicine), whereas electroacupuncture is clinically based on meridian/energy theories (e.g., Chinese medicine). In addition, the targeted points are also different since the needles are inserted into the muscle, joint, or ligament with electrical dry needling, whereas the needles are inserted into acupuncture points in electroacupuncture. The electrical current most commonly applied with PENS interventions is biphasic, with frequencies ranging from 2–5 Hz (low-frequency) to 80–100 Hz (high-frequency) and pulse widths ranging from 250 to 500 ms depending on the effects desired. It is supported that low-frequency electrical stimulation produces an activation of μ and δ opioid receptors, whereas high-frequency electrical stimulation activates δ and k opioid receptors [12].

A meta-analysis investigating the effects of PENS on musculoskeletal chronic pain found low-quality evidence supporting its effect on pain and function [13]. This review found that most published studies using PENS approaches target muscle, tendon, or ligament, but not nerve, tissue [13]. A recent scoping review found a small number of studies investigating PENS specifically targeting nerve tissues [14]. In fact, only two case series have described the effects of PENS in a sample of subjects with shoulder pain after rotator cuff repair [15] or with subacromial impingement syndrome [16]. No clinical trial has investigated the effects of PENS in patients with shoulder pain. Since exercise exhibits strong evidence for managing pain and related disability in shoulder pain, we conducted a randomized clinical trial investigating the effects on pain and related disability of adding PENS targeting the axillary and suprascapular nerves into an exercise program in patients with subacromial pain syndrome. Further, since psychological factors are associated with clinical outcomes in individuals with chronic shoulder pain [17,18], we also investigated the effects of PENS and exercise on anxiety and depressive symptoms, as well as sleep quality. Therefore, the aim of this clinical trial was to investigate the effects on clinical and psychological outcomes of adding four sessions of PENS targeting the suprascapular and axillar nerves to an exercise program in individuals with subacromial pain syndrome. We hypothesized that adding PENS to an exercise program leads to better clinical and psychological outcomes than the application of exercise alone in individuals with subacromial pain syndrome.

## 2. Methods

### 2.1. Study Design

This randomized, parallel-group clinical trial compared the inclusion of PENS into an exercise program for subacromial pain syndrome. The primary endpoint was a three-month improvement in function and related disability. Secondary outcomes included shoulder pain and psychological aspects, such as anxiety levels, depressive symptoms, and sleep quality. This study adhered to the Consolidated Standards of Reporting Trials (CONSORT) extension for clinical trials [19].

The design was approved by the Local Review Board of Universidad Rey Juan Carlos (URJC 0609202218622) and the trial was registered (ClinicalTrials.gov: NCT06158568).

### 2.2. Participants

Consecutive subjects with shoulder pain referred to a local physical therapy clinic at the Universidad Rey Juan Carlos, Madrid (Spain) were screened for eligibility criteria. The subjects included presented with unilateral shoulder pain of non-traumatic origin lasting for at least 3 months and intensity of pain of at least 3 points on an 11-point numerical pain rate scale (NPRS). Further, individuals should report a positive painful arc test during shoulder abduction (+LR 3.7) [20] and a positive Hawkins test (sensitivity 0.58, specificity 0.67) during clinical examination [21]. Participants were excluded if they (1) reported bilateral shoulder pain; (2) were younger than 18 years or older than 65 years old; (3) had a previous history of shoulder traumatism, fracture, or dislocation; (4) had a previous diagnosis of cervical radiculopathy or myelopathy; (5) had received previous interventions with steroid injections in the shoulder; (6) had a diagnosis of comorbid medical conditions, e.g., fibromyalgia syndrome; (7) had a previous history of shoulder or neck surgery; (8) had received any type of therapy in the shoulder area the previous year; or (9) reported a fear of needles. All participants signed an informed consent prior to their inclusion in this study.

Clinical data of all participants, including location, intensity, and duration of shoulder pain symptoms, aggravating and relieving factors, and previous treatments, were collected. Outcomes were assessed at baseline (pre), one week after the last treatment (post), and one and three months after the end of treatment.

### 2.3. Randomization and Masking

Patients were randomly assigned to receive exercise alone, exercise plus PENS, or exercise plus placebo PENS. Concealed allocation was conducted with a computer-generated randomized table of numbers created by an experienced statistician who did not participate in the trial. Individual and sequentially numbered index cards with random assignment were folded and placed in sealed opaque envelopes. An external researcher not involved in data collection opened the envelope and proceeded with treatment allocation.

### 2.4. Exercise Program

All groups received the same exercise program. The exercise program was taught by an experienced physical therapist in the first session and monitored in subsequent sessions. Since no consensus exists on the dosage and type of exercises that should be applied in subacromial pain syndrome, we included a total of six exercises focusing on supraspinatus, infraspinatus, and scapular stabilizer musculature. Each exercise was adapted to the clinical situation of the patient and performed for 3 sets × 10 repetitions. Participants were asked to perform the exercises on an individual basis twice every day for 3 weeks.

### 2.5. Ultrasound-Guided Percutaneous Nerve Stimulation

Participants were placed in prone positions with their shoulders at 90° of abduction and their elbows flexed. An ultrasound Samsung HS50 (Samsung^®^, Seoul, Republic of Korea) with a linear array transducer LA3-14AD was used for the localization of the suprascapular and the axillar nerves in the affected shoulder.

**Figure 1:**
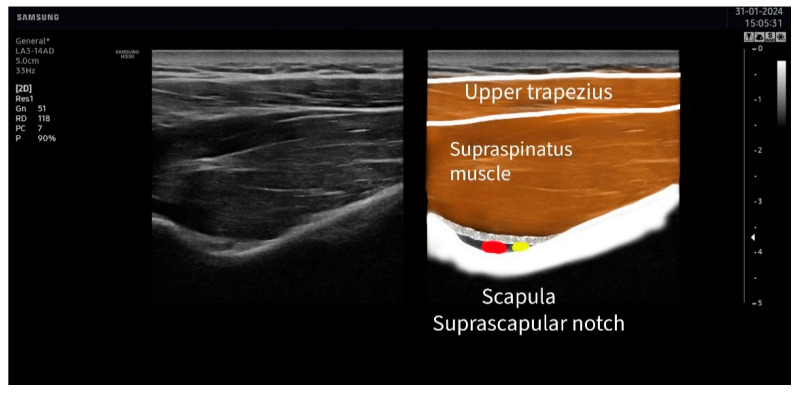
Ultrasound identification of the suprascapular nerve (in yellow) lateral to the artery (in red) at the suprascapular notch. The imaging shows the upper trapezius muscle as the most superficial muscle located over the supraspinatus muscle. The suprascapular notch is located in the deepest part of the suprascapular fossa.

**Figure 2:**
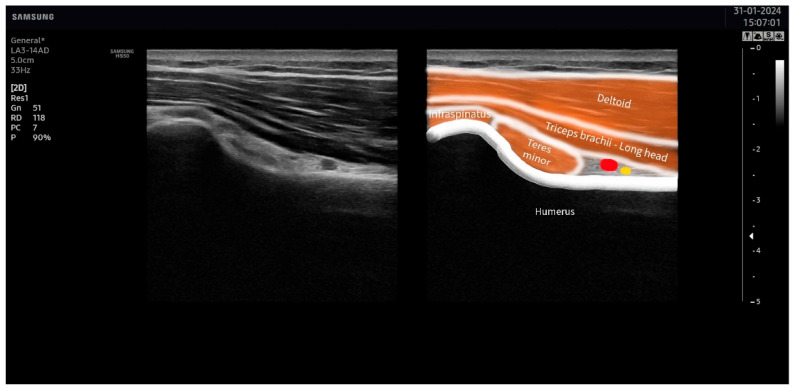
Ultrasound identification of the axillar nerve (in yellow) at the superior border of the quadrangular space under the area between the teres minor and the long head of the triceps muscles. The artery is identified in red.

The suprascapular nerve was identified at the suprascapular notch in the supraspinatus fossa (Figure 1), and the axillar nerve was identified at the superior border of the quadrangular space, between the teres minor and the long head of the triceps muscle (Figure 2).

**Figure 3:**
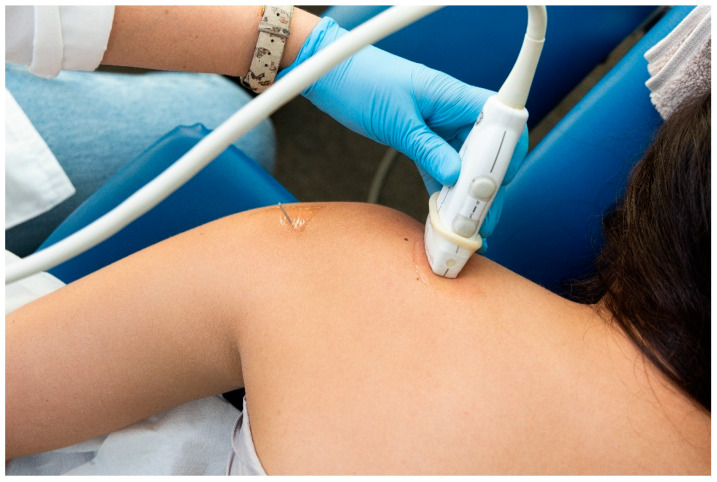
Placement of the ultrasound probe for identification of the suprascapular nerve.

**Figure 4:**
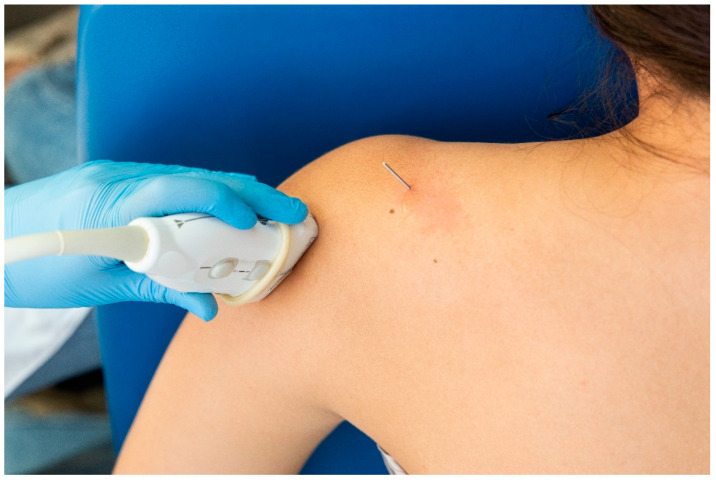
Placement of the ultrasound probe for identification of the axillar nerve.

For the identification of the suprascapular nerve, the US probe was placed transversally to the suprascapular fossa (Figure 3), whereas for the identification of the axillar nerve, the US probe was placed longitudinally to the posterior deltoid muscle but transversally to the quadrangular space (Figure 4).

**Figure 5:**
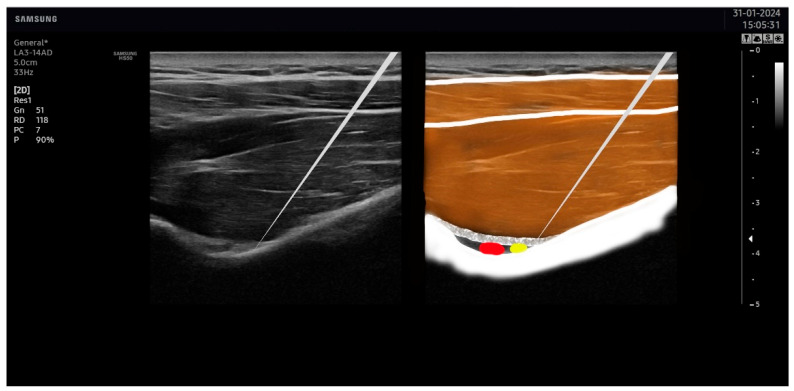
Needle placement on the suprascapular nerve. The tip of the needle was placed as close as possible to the suprascapular nerve (in yellow), avoiding the artery (in red).

**Figure 6:**
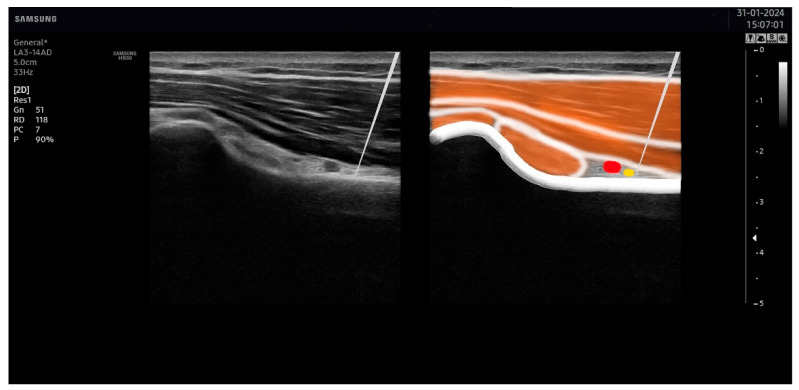
Needle placement on the axillar nerve. The tip of the needle was placed as close as possible to the axillar nerve (in yellow), avoiding the artery (in red).

Once these points were visualized with the ultrasound, the overlaying skin was disinfected with antiseptic (Cutasept, Hartmann), and a solid filiform needle (0.30 × 50 mm AguPunt, Spain) was inserted “in-plane” until the tip was located as close as possible to the epineurium of the suprascapular (Figure 5) or the axillar (Figure 6) nerve was reached.

To confirm that each nerve was targeted, a Pointer Plus stimulator (Goldberg International Enterprises Ltd., Kowloon, Hong Kong) was applied over the needle to stimulate the innervated muscles by means of 2 or 3 electrical pulses at a frequency of 10 Hz and 2.5–3 mA of intensity. Needle placement was considered accurate when the application of the Pointer Plus stimulation should produce a visible muscle contraction of the innervated muscles of the axillar (deltoid and teres minor muscles) or suprascapular (supraspinatus and infraspinatus muscles) nerve.

**Figure 7:**
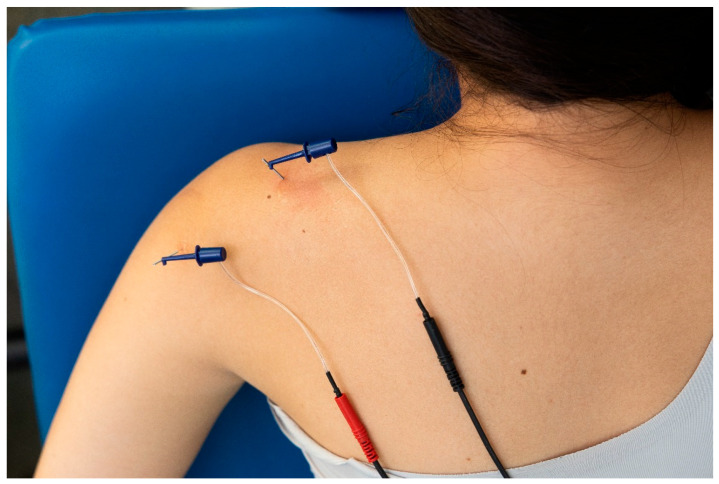
Application of percutaneous nerve stimulation (PENS) on the suprascapular and axillar nerves on a patient with shoulder pain.

Patients allocated to the exercise plus PENS received four sessions (once per week) of 30 min duration of ultrasound-guided percutaneous electrical nerve stimulation targeting the suprascapular and axillar nerves. For PENS treatment, two needles, one in the axillar nerve and the other in the suprascapular nerve, were connected to an electro-stimulator (ENRAF NONIUS endomed 484, Madrid, Spain). Then, a biphasic square wave electrical current at a 2 Hz frequency with a 250-microsecond pulse width was applied (Figure 7). The intensity of the electrical current was increased to induce an involuntary contraction of the innervated muscles below the pain threshold.

### 2.6. Placebo Ultrasound-Guided Percutaneous Nerve Stimulation

Patients allocated to the exercise plus placebo PENS received four sessions (one per week) of ultrasound-guided placebo PENS targeting the suprascapular and axillar nerves for 30 min. The position of the patient, location of the suprascapular/axillar nerves, and location of the needle insertion were the same in the exercise plus PENS group but without the application of any electrical current. The needles were connected to the electro-stimulator mimicking the experimental group, but in a different channel, so the light of the device was brightening, and the patient could hear the final whistle. This placebo method has been used in other studies with sham percutaneous stimulation [22].

### 2.7. Primary Outcome

The primary outcome included related disability assessed with the Spanish version of the Disabilities of the Arm, Shoulder, and Hand (DASH) questionnaire, which has shown high internal consistency (Cronbach α: 0.96) and excellent test–retest reliability (r: 0.96) [23]. The DASH includes 21 items assessing problems experienced during the preceding week of performing physical activities, 5 items evaluating the severity of different pain symptoms, and 4 items assessing the effects of pain on social activities, work, and sleep. Each item is answered on a 5-point Likert scale (1: no problem, no symptom, no impact; 5: impossible to perform, extremely severe symptom, high impact). The total score ranges from 0 to 100 points, where a higher score reflects greater pain-related disability [24]. It has been reported that the minimal clinical important difference (MCID) for the DASH is 10.8 points [25].

### 2.8. Secondary Outcomes

The intensity of shoulder pain in the preceding week was assessed with an 11-point NPRS (0: no pain; 10: maximum pain) [26]. The MCID for the NPRS in people with shoulder pain has been estimated to be 1.1 points [27].

The Spanish version of the Hospital Anxiety and Depression Scale (HADS) was used for assessing the presence of anxiety levels (HADS-A) and depressive symptoms (HADS-D) [28]. Each scale includes 7 items, each one with scores ranging from 0 to 3 points [29]. The total score of each subscale (HADS-A, HADS-D) is calculated from the sum of each item (some items are inversely scored), leading to a total score ranging from 0 to 21 points [29]. The psychometric properties of both scales have been shown to be good [30].

The Spanish version of the Pittsburgh Sleep Quality Index (PSQI) was used for assessing the quality of sleep [31]. The PSQI provides a total score ranging from 0 to 21 points based on 19 questions evaluating different aspects of sleep, e.g., usual bedtime, wake-up time, number of hours slept, and time needed to fall asleep [32].

### 2.9. Treatment Side Effects

Patients were asked to report any adverse event that they experienced either after the intervention or during any other part of this study. In the current study, an adverse event was defined as sequelae with any symptom perceived as distressing to the patient and requiring further treatment.

### 2.10. Sample Size Calculation

The sample size was calculated by using the G*Power software 3.1.9.2 (Heinrich, Heine University, Düsseldorf, Germany). An a priori power analysis with an F test ANOVA for repeated measures was conducted, with an alpha level (α) of 0.05 and a desired statistical power (β) of 90%. The calculation was based on detecting differences of a 10.8-point (MCID) on the main outcome between the exercise plus PENS group and the remaining groups [25], assuming a standard deviation of 10 points. Based on these assumptions, a sample size of 19 subjects per group was obtained.

### 2.11. Statistical Analysis

Statistical analyses were performed using SPSS Statistics v.25 for Windows and were conducted according to the intention-to-treat analysis principle for patients in the group to which they were allocated. The normal distribution of the variables was assessed with the Kolmogorov–Smirnov test (*p* > 0.05). Between-group differences at baseline were compared with Fisher’s exact test for categorical data or one-way analysis of variance (ANOVA) for continuous data. Our evaluation included mixed-model repeated measured analyses of covariance (ANCOVA), with time (baseline, after, one month, three months) as the within-subjects factor, group (exercise, PENS, placebo PENS) as the between-subjects factor, and adjusted for baseline outcomes for evaluating between-group differences in all outcomes. Post hoc pairwise comparisons were analyzed using Student’s *t*-tests for independent samples. The partial eta squared (n^2^_p_) was calculated to estimate the effect size according to those recommendations in repeated measures models [33]. An effect size of 0.01 was considered small, 0.06 was considered medium, and 0.14 was considered large in partial eta squared. An alpha level of 0.05 and 95% confidence intervals (CI) were assumed for all analyses.

## 3. Results

**Figure 8:**
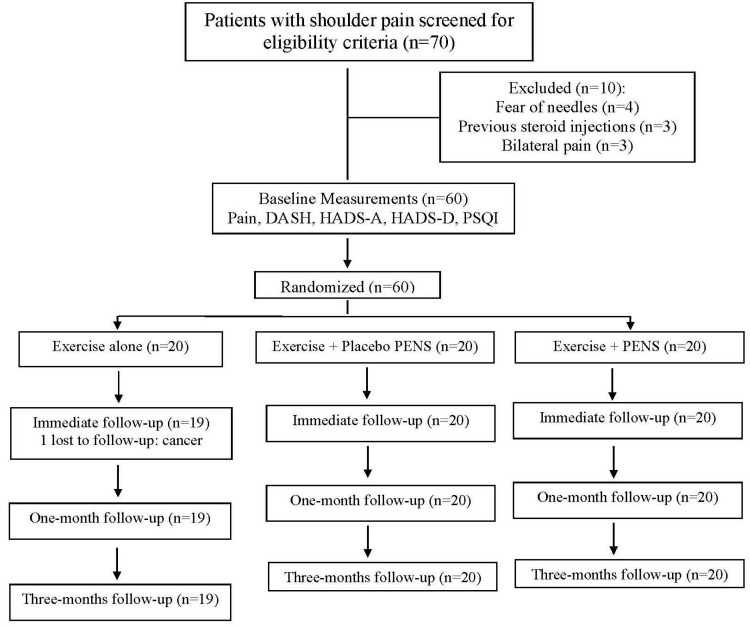
Flow diagram of patients throughout the course of this study.

Between 10 December 2023 and 15 January 2024, 70 consecutive subjects reporting shoulder pain were screened for eligibility criteria. Sixty (85%) satisfied all the criteria, agreed to participate, and were randomly allocated into exercise alone (n = 20), exercise plus PENS (n = 20), or exercise plus placebo PENS (n = 20) groups. One patient allocated to the exercise alone group was lost because they developed cancer. Figure 8 provides a flow diagram of patient recruitment and retention. Randomization resulted in similar baseline features for all variables (Table 1). All patients assigned to the exercise plus PENS group experienced slight soreness after the first session, which resolved spontaneously within 24–36 h. No adverse events were reported by the participants during this study.

### 3.1. Clinical Outcomes

Adjusting for baseline outcomes, the mixed-model ANCOVA revealed a significant Group*Time interaction (F = 3.370; *p* = 0.04; n^2^_p_ = 0.109) for shoulder pain but not for related disability (F = 0.292; *p* = 0.748; n^2^_p_ = 0.011). Post hoc analyses revealed that patients receiving PENS plus exercise exhibited greater improvement in shoulder pain at one month follow-up period than those in the exercise (Δ −1.2, 95%CI −2.3 to −0.1, *p* = 0.045) or the placebo PENS plus exercise (Δ −1.3, 95%CI −2.5 to −0.1, *p* = 0.04) groups (Table 2). No significant differences among the groups at three months follow-up for shoulder pain were identified. A significant time effect (F = 25.097; *p* < 0.001; n^2^_p_ = 0.550) for related disability (DASH) was observed; all groups experienced similar improvements in related disability (DASH score) throughout all follow-up periods (Table 2).

No significant effect of sex or age was found for either pain intensity (sex: F = 1.061; *p* = 0.491; n^2^_p_ = 0.05; age: F = 1.222; *p* = 0.628; n^2^_p_ = 0.035) or related disability (sex: F = 0.917; *p* = 0.514; n^2^_p_ = 0.045; age: F = 1.361; *p* = 0.645; n^2^_p_ = 0.03).

### 3.2. Psychological Outcomes

Table 3 shows changes in psychological outcomes. Adjusting for baseline outcomes, the mixed-model ANCOVA did not reveal significant Group*Time interactions for anxiety levels (HADS-A: F = 0.780; *p* = 0.463; n^2^_p_ = 0.027), depressive symptoms (HADS-D: F = 0.559; *p* = 0.575; n^2^_p_ = 0.02), or sleep quality (PSQI: F = 0.294; *p*= 0.747; n^2^_p_ = 0.01). A significant time effect was observed for all psychological outcomes (HADS-A: F = 5.928; *p* = 0.01; n^2^_p_ = 0.109; HADS-D: F = 6.370; *p* = 0.012; n^2^_p_ = 0.108; PSQI: F= 16.722; *p* < 0.001; n^2^_p_ = 0.236); all groups experienced similar improvements in anxiety (HADS-A), depressive symptoms (HADS-D), and sleep quality (PSQI) throughout all follow-up (Table 3). The effect size was large for changes in sleep quality and moderate–large for anxiety levels or depressive symptoms.

No significant effect of sex or age was found for anxiety levels (sex: F = 0.085; *p* = 0.819; n^2^_p_ = 0.007; age: F = 0.704; *p* = 0.758; n^2^_p_ = 0.005), depressive symptoms (sex: F = 0.140; *p* = 0.772; n^2^_p_ = 0.003; age: F = 1.184; *p* = 0.636; n^2^_p_ = 0.03), or sleep quality (sex: F = 0.009; *p* = 0.940; n^2^_p_ = 0.001; age: F = 0.249; *p* = 0.947; n^2^_p_ = 0.001).

## 4. Discussion

This is the first study investigating the effect of adding PENS targeting the nerve tissues to an exercise program for the treatment of subacromial pain syndrome. This randomized clinical trial found that adding four sessions of PENS targeting the suprascapular and axillar nerves into an exercise program did not result in better outcomes for clinical, e.g., related disability or pain, and psychological, e.g., anxiety levels, depressive symptoms, or sleep quality, outcomes in individuals with subacromial pain syndrome at one- and three-month follow-ups. The current results would agree with a recent meta-analysis concluding that adding other interventions e.g., manual therapy, to an exercise program is not more effective than exercise alone for the management of rotator cuff-related shoulder pain [34].

### 4.1. Effects of PENS on Pain and Related Disability

The use of exercise for the management of patients with subacromial pain syndrome is further supported in the literature [10]. Our study found that the three groups experienced clinical improvements in pain and related disability, independently of the inclusion or not of four sessions of PENS targeting the suprascapular or axillar nerve. In fact, within-group change scores and their 95% confidence intervals surpassed the MCID of 10.8 points for related disability [25] and of 1.1 points for shoulder pain [26], supporting the clinical effect of the exercise program. Nevertheless, the current results should be considered according to the dosage of exercise, exercise loading strategy, or those specific exercises included in the current trial. Thus, the question to be answered is which treatment strategies can be combined to promote the effects of exercise in this condition.

The novelty of this trial was the application, for the first time, of PENS targeting the axillar and suprascapular nerves for the management of subacromial pain syndrome. We found that individuals receiving four sessions of PENS targeting the suprascapular or axillar nerve plus exercise only exhibited higher improvements in pain at one month follow-up than those who received the exercise program alone and those receiving exercise plus placebo PENS. In this case, between-group mean change scores, but not their 95% confidence intervals, slightly surpassed the MCID of 1.1 points for shoulder pain [26], supporting that the clinical relevance of PENS is small with the current dosage. In addition, the inclusion of four sessions of PENS targeting the suprascapular or axillar nerves did not produce a significant additional effect of exercise on related disability.

No previous clinical trial has investigated the use of PENS targeting the nerve tissue for the management of chronic should pain; however, there are some studies investigating the effects of other forms of PENS, e.g., electrical dry needling or electroacupuncture, in this condition. Current results agree with a previous study showing that adding electroacupuncture to an exercise program did not exert a positive effect on subacromial pain syndrome [35] but disagree with another trial showing that the use of electrical dry needling, combined with manual therapy, was effective in individuals with shoulder pain up to three months after [36]. Differences in study populations, the dosage of exercise programs, the frequency and type of the PENS treatment, or other intervention approaches could explain the discrepancies between studies. For instance, Lewis et al. included individuals with full thickness or massive irreparable rotator cuff tears [35], conditions that were excluded in our study. It would be expected that conservative interventions applied to patients will have less clinical effect on patients exhibiting tissue damage, such as tendon full thickness. Nevertheless, the most important difference among these trials to discuss is the experimental approach. Lewis et al. [35] applied electroacupuncture, whereas Dunning et al. [36] used electrical dry needling. Accordingly, needles were inserted into muscles, joints, or ligaments with electrical dry needling [36] or into acupuncture points [35]. In the current trial, the needles were placed close to the nerve tissue, making a real percutaneous nerve stimulation. Thus, the number of needles inserted in our study was just two, one on each nerve, whereas the number of needles inserted in both Lewis et al. [35] and Dunning et al. [36] was higher. Additionally, the dosage of the PENS intervention was also different since Lewis et al. [35] applied six treatment sessions of electroacupuncture, whereas Dunning et al. [36] applied twelve sessions of electrical dry needling. It seems that the dosage of our study (four sessions) and the Lewis et al. [35] study (six sessions) could have been small for obtaining clinical effects, and a larger number of sessions could be needed.

### 4.2. Effects of PENS on Psychological Aspects

Another aspect of this trial was the inclusion of psychological variables since emotional stress has been associated with higher levels of disability in the long term [37] and also with worse clinical outcomes after physical therapy treatment in individuals with chronic shoulder pain [17,18]. Interestingly, Smedbråten et al. observed that the presence of higher emotional distress in individuals with shoulder pain was associated with higher pain intensity, but not with more related disability, after physical therapy management [18]. Our trial did not find significant changes in psychological variables after the application of exercise combined or not with PENS. It is probable that the lower levels of anxiety and depressive levels, as well as the absence of poor sleepers, based on the lower scores found in the HADS and PSQI, in our sample of patients with shoulder pain, would explain these results. Another explanation can also be related to the fact that the effect of hands-on intervention, e.g., manual therapy or PENS, is small in pain-related fear outcomes [38]. It is probable that, in those patients with shoulder pain exhibiting higher levels of stress, the inclusion of psychological interventions into a multimodal treatment approach must be included.

### 4.3. Underlying Mechanisms of PENS Targeting the Nerve Tissue

Although the current trial did not find a clinical effect of the application of PENS when combined with exercise for subacromial pain syndrome, some hypotheses explaining its potential mechanisms can be discussed. The underlying mechanisms of PENS are based on the following two points: (1) the use of electrical current and (2) its application through a needle.

Langevin et al. found that the use of electrical current (electroacupuncture) exerted a greater analgesic effect than just the use of a solid needle (acupuncture) for chronic pain [39]. A recent meta-analysis found that combining needling interventions with an electrical current (electrical dry needling) seems to be more effective than the application of just the needle (dry needling) in treating musculoskeletal shoulder pain [40]. In fact, the clinical benefit of electrical current on chronic pain conditions is supported by the application of transcutaneous nerve stimulation (TENS). Thus, moderate evidence suggests that TENS can reduce pain intensity in musculoskeletal pain conditions [41]. Thus, moderate evidence also suggests that the effects of TENS for promoting analgesia in acute/chronic pain conditions are related to a reduction in primary (peripheral mechanism) and secondary (central mechanism) hyperalgesia [42]. It is suggested that these sensitization effects of the electrical current depend on the frequency of application, high frequency (e.g., 80–100 Hz) and low frequency (e.g., 2–10 Hz), in which the activation of endogenous opioid receptors depends on the stimulation received. In this study, we applied a low-frequency protocol (2 Hz) at an intensity producing muscle contraction. By using a similar protocol, i.e., low frequency for long period of time, Beltrá et al. found that this protocol was able to increase the electrical motor threshold but reduced motor recruitment without eliciting an hypoalgesic effect in health subjects [43]. Accordingly, we do not know if a different protocol including higher frequencies would lead to different results.

The second topic to discuss is the application of the electrical current through a needle or not. A recent meta-analysis found low-quality evidence suggesting that PENS is more effective for reducing pain intensity than TENS, but the differences were not clinically relevant [44]. In fact, both PENS and TENS have a mild to moderate immediate effect on local (peripheral) and remote (central) mechanical hyperalgesia in patients with chronic pain of musculoskeletal origin, although further studies are clearly needed to confirm these conclusions [45].

### 4.4. Limitations

The results of the current clinical trial should be considered according to its strengths and limitations. The major strengths of this trial investigating the effects of PENS targeting the nerve tissue in patients with subacromial pain syndrome are (1) the inclusion of a placebo PENS intervention; (2) this study adhered to CONSORT guidelines; (3) the use of concealed allocation; and (4) the statistical analysis was conducted following the intention-to-treat principle. Potential limitations include (1) all patients were recruited from a single clinic which may decrease the generalization of the results. Therefore, future multicenter trials controlling for clinician effects (cluster effects) might enhance the generalizability of the results; (2) although we included a placebo group, we did not include a wait-and-see or no-intervention group; hence, we cannot exclude that the improvements experienced are due to the natural history of the condition or placebo; (3) individuals allocated to the PENS group received just four sessions based on the author clinical experience since no scientific data is available determining the dose or the frequency of the interventions; therefore, we do not know if a higher number or a higher frequency of sessions would result in larger differences; and (4) we only assessed mid-term effects (3 months after); we do not know if changes will be similar with longer follow-up periods. Accordingly, future multicenter clinical trials including a large number of sessions of PENS, probably 10–12 sessions combined with an exercise program, also of 12 weeks, could identify the potential clinical effects of this intervention in patients with chronic shoulder pain. In addition, it is also possible that not all patients with shoulder pain will benefit from the application of PENS targeting nerve tissues. It would be conceivable that patients exhibiting nerve mechanical sensitivity would experience better clinical outcomes with PENS than patients for whom the nociceptive source would be different, e.g., tendon or muscle. Future studies identifying the clinical features of “responders” would help to confirm or refute the clinical effects of the proposed intervention.

## 5. Conclusions

In conclusion, our data indicate that the inclusion of four sessions (once per week) of PENS targeting the suprascapular and axillar nerves into an exercise program did not result in better outcomes in individuals with our sample of patients with subacromial pain syndrome at one and three months after treatment. The inclusion of PENS into an exercise program resulted in a greater improvement in shoulder pain at one month, but not three months, after the intervention, but changes were not clinically meaningful. No changes in psychological variables were found either. These results should be considered according to the limitations of the current clinical trial. Future multicenter studies including a wait-and-see group, a different dosage of the intervention, and longer follow-up periods are now needed to confirm or refute current results.

## Figures and Tables

**Table 1:** Baseline characteristics by treatment assignment.

	Exercise (n = 19)	PENS + Exercise (n = 20)	Placebo PENS + Exercise (n = 20)
Gender (male/female)	7 (37%)/12 (63%)	8 (40%)/12 (60%)	7 (35%)/13 (65%)
Age (years)	45.5 ± 11	45 ± 11	46 ± 10
Months with pain	15.2 ± 9.6	16.2 ± 8.5	15.0 ± 7.0
Side of the symptoms n (%) Right side Left side	10 (58%) 7 (42%)	13 (59%) 9 (41%)	11 (55%) 9 (45%)
Mean pain intensity (NPRS, 0–10)	5.6 ± 2.2	5.25 ± 1.5	5.8 ± 2.2
DASH (0–100)	73.5 ± 23.0	66.0 ± 20.5	73.0 ± 20.0
HADS-A (0–21)	6.8 ± 4.2	7.3 ± 3.5	9.0 ± 4.5
HADS-D (0–21)	3.0 ± 2.8	3.3 ± 2.4	4.8 ± 2.6
PSQI (0–21)	9.3 ± 3.8	8.5 ± 3.2	9.3 ± 3.3

NPRS: Numerical Pain Rate Scale; DASH: Disabilities of the Arm, Shoulder, and Hand; HADS: Hospital Anxiety and Depression Scale (A: anxiety, D: depression); PSQI: Pittsburgh Sleep Quality Index.

**Table 2:** Clinical outcomes before and one and three months after intervention by randomized treatment assignment.

Outcome Group	Pre-Intervention	Post-Intervention	1 Month	3 Months
Mean intensity of shoulder pain (NPRS, 0–10)				
Exercise	5.6 ± 2.2 (4.7, 6.5)	4.7 ± 2.7 (3.7, 5.7)	3.6 ± 2.2 (2.7, 4.5)	2.5 ± 2.1 (1.7, 3.3)
PENS + Exercise	5.25 ± 1.5 (4.4, 6.1)	3.8 ± 1.8 (2.9, 4.7)	2.3 ± 1.7 (1.5, 3.1)	2.8 ± 1.1 (2.1, 3.5)
Placebo PENS + Exercise	5.8 ± 2.2 (5.8, 6.6)	4.4 ± 2.1 (3.4, 5.4)	3.5 ± 1.9 (2.7, 4.3)	2.7 ± 1.9 (1.9, 3.5)
Disabilities of the Arm, Shoulder and Hand (DASH, 0–100)				
Exercise	73.5 ± 23.0 (63.1, 83.9)	56.9 ± 17.1 (49.1, 64.7)	51.4± 20.7 (43.4, 59.4)	48.5 ± 16.0 (41.1, 55.9)
PENS + Exercise	66.0 ± 20.5 (56.7, 75.3)	55.9 ± 15.8 (49.2, 62.6)	50.1 ± 12.9 (43.2, 57.0)	48.7 ± 13.4 (42.3, 55.1)
Placebo PENS + Exercise	73.0 ± 20.0 (63.5, 82.5)	58.9 ± 14.5 (51.9, 65.9)	51.9 ± 14.7 (44.7, 59.1)	48.2 ± 15.5 (41.6, 54.8)

NPRS: Numerical Pain Rate Scale.

**Table 3:** Psychological outcomes before and one and three months after intervention by randomized treatment assignment.

Outcome Group	Pre-Intervention	Post-Intervention	1 Month	3 Months
Hospital Anxiety and Depression Scale—Anxiety (HADS-A, 0–21)				
Exercise	6.8 ± 4.2 (4.9, 8.7)	6.3 ± 3.9 (4.4, 8.2)	4.7 ± 3.7 (3.0, 6.4)	4.1 ± 3.4 (2.4, 5.8)
PENS + Exercise	7.3 ± 3.5 (5.7, 8.9)	6.2 ± 3.4 (4.5, 7.9)	5.7 ± 3.1 (4.3, 7.1)	5.2 ± 2.9 (3.7, 6.7)
Placebo PENS + Exercise	9.0 ± 4.5 (7.4, 10.6)	7.9 ± 4.4 (6.1, 9.7)	6.3 ± 3.7 (4.8, 7.8)	6.3 ± 3.7 (4.8, 7.8)
Hospital Anxiety and Depression Scale—Depression (HADS-D, 0–21)				
Exercise	3.0 ± 2.8 (1.9, 4.1)	2.7 ± 2.7 (1.2, 4.2)	2.3 ± 3.0 (0.9, 3.7)	2.1 ± 2.7 (0.7, 3.5)
PENS + Exercise	3.3 ± 2.4 (2.2, 4.4)	3.0 ± 2.7 (1.7, 4.3)	2.8 ± 2.7 (1.6, 4.0)	2.8 ± 2.8 (1.6, 4.0)
Placebo PENS + Exercise	4.8 ± 2.6 (3.6, 6.0)	3.4 ± 2.5 (2.0, 4.8)	3.6 ± 3.0 (2.3, 4.9)	3.4 ± 3.0 (2.2, 4.6)
Pittsburgh Sleep Quality Index (PSQI, 0–21)				
Exercise	9.3 ± 3.8 (7.5, 11.1)	8.5 ± 4.0 (6.8, 10.2)	8.1 ± 4.0 (6.4, 9.8)	7.7 ± 3.7 (6.2, 9.2)
PENS + Exercise	8.5 ± 3.2 (7.1, 9.9)	8.2 ± 2.8 (6.8, 9.6)	7.0 ± 2.5 (5.6, 8.4)	7.1 ± 2.2 (5.9, 8.3)
Placebo PENS + Exercise	9.3 ± 3.3 (7.8, 10.8)	8.4 ± 3.0 (7.0, 9.8)	7.4 ± 3.3 (5.9, 8.9)	7.1 ± 2.8 (5.8, 8.4)

## Data Availability

The data presented in this study are available on request from the corresponding author.
